# Supplementary material for: Methylase-assisted subcloning for high throughput BioBrick assembly
Source: PeerJ. 2020 Sep 11;8:e9841. doi: 10.7717/peerj.9841 (PMC7489255; doi:10.7717/peerj.9841)
Supplement: Supplemental Information 1 [file peerj-08-9841-s001.docx]

Supplemental files for article submitted to PeerJ, July 7, 2020

Methylase-assisted subcloning for high throughput BioBrick assembly

Ichiro Matsumura^1^

^1^Department of Biochemistry, Emory University School of Medicine, Atlanta, Georgia, USA

Corresponding author:

Ichiro Matsumura

1510 Clifton Road NE, room 4119

Atlanta, GA 30322, USA

e-mail address: imatsum@emory.edu

tele: (404) 727-5625

**Supplemental Results**

Tip Snip subcloning

I previously described “Tip Snip” subcloning to circumvent preparative agarose gel electrophoresis and gel extraction (Matsumura 2017). Briefly, the undesired donor plasmid is shortened slightly by the action of an additional restriction enzyme that leaves it with a different sticky end. The short restriction fragments in both digests are inactivated via denaturation and annealing to complementary synthetic oligonucleotides and size selection spin column chromatography. Ligations include a pair of complementary oligonucleotides that prevent self-ligation of incompletely digested, unwanted donor plasmid. Transformation of chemically competent *E. coli* cells with the ligation reactions produced 384 ± 61 pink cfu/ng, a three-fold improvement over the conventional approach, with less background in insert only ligation controls (9 ± 6 white cfu/ng = 2.3%, three-fold more accurate that of gel purified DNA, Table 1). These results are consistent with the hypothesis that agarose contains inhibitors of T4 DNA ligase, and that restriction enzymes can distinguish desired and undesired restriction fragments more efficiently than does gel electrophoresis.

3A assembly

The 3A BioBrick assembly technique (Shetty et al. 2011) is currently recommended along with Loop Assembly (Pollak et al. 2019) by organizers of the International Genetically Engineered Machine (iGEM) competition. Briefly, both BioBrick inserts are cut from their respective donor plasmids (Figure 6 and S10) and ligated into a third recipient plasmid that encodes a selectable marker different than those on the donors (Figures S11 and S12). This approach circumvents gel purification but has never been efficient in my hands. Transformation of competent *E. coli* with 3A ligation products produced only 4 ± 1 pink colonies per ng, mixed with a similar number (5 ± 2) of white colonies (Table 1). The roughly equal numbers of pink and white colonies indicate that rigorous screening of the generally uncolored colonies produced by 3A assembly would normally be required.

The inefficiency and inaccuracy of 3A assembly is not surprising when all reaction intermediates and products are considered. Cartoons that depict 3A assembly typically only show the desired inserts and recipient plasmid, but the undesired donor plasmids and stuffer fragments are still T4 DNA ligase substrates (Figure S10). The lacI-Ptac-lacO restriction fragment, for example, is supposed to ligate to the pUC-cat fragment because both have EcoRI sticky ends. Yet each of these fragments could also ligate to other copies of themselves, the EcoRI-cut donor plasmid or the EcoRI-cut stuffer. A relatively small fraction of ligation products (2/5 * 2/5 * 2/6 = 8/150 = 5.3%) will be those that are desired; any incorrect product that includes the pUC-cat recipient plasmid will enable a transformed cell to form a colony on LB-chloramphenicol plates (Figure S11), as noted by the developers of the 3A assembly method (Shetty et al. 2011).

**Supplemental Figures**

**Figure S1. Digested model plasmids for 2RM assembly**

Model plasmids 1 (lacI-Ptac-lacO-pUC, methylated at its XbaI site) and 2 (tagRFP-pUC, methylated at its SpeI site, Figure 3) were mixed together and digested with both XbaI and SpeI-HF. Each plasmid was protected from the action of one restriction enzyme and susceptible to the other (Figure 5).

**Figure S2. Homodimeric ligation products in 2RM assembly of model plasmids**

Linearized plasmids 1 and 2 (Figure S1) can ligate to other copies of themselves in two possible orientations. All these ligation products remain susceptible to digestion by XbaI or SpeI-HF. The head to head dimers form inverted repeats that are unlikely to replicate in *E. coli*.

**Figure S3. Heterodimeric ligation products in 2RM assembly of model plasmids**

Linearized plasmids 1 and 2 (Figure S1) can ligate to each other in two possible orientations, both of which are resistant to XbaI or SpeI-HF. Both products can transform *E. coli* efficiently so colony screening will generally be necessary to distinguish desired clones (left) from those that are not (right). The desired construct (left) encodes correctly assembled inserts but also contains a redundant copy of the plasmid origin and selectable marker.

**Figure S4. Digests of model plasmids for 4R/2M (PstI) assembly**

In 4R/2M (PstI) BioBrick assembly (Figure 6), the donor plasmid, tagRFP-pUC (Figure 3), is purified from *E. coli* expressing M.Ocy1ORF8430P and subsequently digested with XbaI and PstI. The recipient plasmid, lacI-Ptac-lacO-pUC, is protected by M.EcoRI and digested with SpeI and PstI.

**Figure S5. Homodimeric ligation products of 4R/2M (PstI) assembly of model plasmids**

The 4R/2M (PstI) restriction fragments (Figure S4) can ligate to other copies of themselves. None of the resulting homodimeric ligation products, however, replicate stably within *E. coli* because inverted repeats destabilize plasmids.

**Figure S6. Heterodimeric ligation products of 4R/2M (PstI) assembly of model plasmids**

Each of the four 4R/2M (PstI) restriction fragments (Figure S5) can ligate to one of two other fragments, creating four distinct ligation products. (Top left) One does not encode a selectable marker or origin of replication. (Top right and Bottom left) Two others remain susceptible to SpeI or EcoRI. (Bottom right) Only the desired ligation product includes a selectable marker and origin of replication and is also resistant to both SpeI and EcoRI. It alone retains its ability to transform *E. coli* after double digestion of 4R/2M (PstI) ligation products with these enzymes.

**Figure S7. Digests of model plasmids for 4R/2M (EcoRI) assembly**

In 4R/2M (EcoRI) BioBrick assembly, the donor plasmid, lacI-Ptac-lacO-pUC (Figure 3), is purified from *E. coli* expressing M.XbaI and subsequently digested with EcoRI-HF and SpeI-HF. The recipient plasmid, tagRFP-pUC, is protected by either M.PstI or M.AvaIII, which recognizes the NsiI site adjacent to PstI, and digested with EcoRI-HF and XbaI.

**Figure S8. Homodimeric ligation products of 4R/2M (EcoRI) assembly of model plasmids**

4R/2M (EcoRI) restriction fragments (Figure S7) ligate to other copies of themselves, although none of the homodimeric ligation products are viable in *E. coli*.

**Figure S9. Heterodimeric ligation products of 4R/2M (EcoRI) assembly of model plasmids**

Each 4R/2M (EcoRI) restriction fragments (Figure S7) can ligate to one of three other fragments, creating six distinct ligation products. Two are the original parental plasmids (Figure 3). Another does not include any selectable marker or origin of replication. (Top right and Bottom left) Two others remain susceptible to XbaI, PstI (if M.PstI was used to protect the recipient plasmid) or NsiI (if M.AvaIII was used). (Bottom right) The desired ligation product is the only viable plasmid resists digestion by both XbaI and PstI (or NsiI).

**Figure S10. 3A restriction fragments**

Double digests of three plasmids (Figure 2) produces six restriction fragments, three of which are desired (inserts 1 and 2, recipient plasmid) and three that are not (donor plasmids 1 and 2, stuffer). Each fragment can ligate to one of four, including another copy of itself (not shown).

**Figure S11. Viable trimeric 3A ligation products**

The six restriction fragments produced during 3A assembly can ligate to each other to produce a variety of products (not shown). Only those that are circular and contain the right selectable marker (chloramphenicol acetyltransferase in this case) are viable. Still, most *E. coli* colonies will carry undesired ligation products (top and bottom left) so colony screening is required to identify the desired construct (bottom right).

**Supplemental References**

Matsumura I. 2017. Semi-automated Tip Snip cloning of restriction fragments into and out of plasmid polylinkers. *Biotechniques* 62:99-106. 10.2144/000114522

Pollak B, Cerda A, Delmans M, Alamos S, Moyano T, West A, Gutierrez RA, Patron NJ, Federici F, and Haseloff J. 2019. Loop assembly: a simple and open system for recursive fabrication of DNA circuits. *New Phytol* 222:628-640. 10.1111/nph.15625

Shetty R, Lizarazo M, Rettberg R, and Knight TF. 2011. Assembly of BioBrick standard biological parts using three antibiotic assembly. *Methods Enzymol* 498:311-326. 10.1016/B978-0-12-385120-8.00013-9
